# Comparative Evaluation of Physicochemical Stability and In Vitro Intestinal Dialysability of Conventional Solid and Liquid Nanoscale Vitamin Premixes in Swine Feed

**DOI:** 10.3390/vetsci13101043

**Published:** 2026-10-03

**Authors:** Lujun Zhang, Yamei Wang, Ruxue Tang, Mingming Tao, Xipan Hou, Zhifang Chu, Weijiang Zheng, Wen Yao

**Affiliations:** 1 Department of Animal Nutrition and Feed Science, College of Animal Science and Technology, Nanjing Agricultural University, Nanjing 210095, China; 2 Sanya Institute of Nanjing Agricultural University, Nanjing Agricultural University, Sanya 572025, China

**Keywords:** dialysability, fat-soluble vitamins, physicochemical stability, swine nutrition, vitamin premix

## Abstract

Fat-soluble vitamins (FSVs) are essential micronutrients in swine nutrition, but their stability can be affected by formulation characteristics, storage conditions, thermal processing, and gastrointestinal environments. This study aimed to compare the physicochemical stability and in vitro intestinal dialysability of vitamins A, D, and E between two non-identical commercial formulations: conventional solid vitamin premixes (SVPs) and liquid nanoscale vitamin premixes (LNVPs) under simulated storage, thermal treatment, gastrointestinal digestion, dialysis, and fecal fermentation conditions. Microscopy revealed irregular solid particles in SVPs and spherical nanoscale structures observed in LNVPs under transmission electron microscopy conditions, with the latter showing improved visual dispersion stability. Vitamin retention decreased progressively during storage, and LNVPs maintained significantly higher relative retention of FSVs than SVPs (*p* < 0.01). After 40 days of storage at 40 °C, LNVPs retained more than 65% of vitamins D and E, whereas SVPs retained less than 50%. Under high-temperature stress simulating feed pelleting, LNVPs also showed higher retention of vitamins D and E than SVPs. In the simulated porcine gastrointestinal digestion model, LNVPs showed lower early apparent disappearance and higher dialysable fractions during the intestinal phase for all three FSVs (*p* < 0.01). A subsequent fecal fermentation assay further revealed formulation-dependent differences in vitamin behavior under simulated colonic conditions. Collectively, LNVPs showed higher relative vitamin retention and greater in vitro dialysability than SVPs under the tested conditions, supporting the potential of LNVPs for improving vitamin preservation in swine feed applications.

## Simple Summary

Vitamins A, D, and E are important micronutrients in swine nutrition; however, their stability during storage, thermal processing, and gastrointestinal conditions may affect their measurable availability. Liquid vitamin formulations based on advanced delivery systems have been developed as alternatives to conventional solid premixes, but comparative information regarding their physicochemical stability and in vitro vitamin behavior remains limited. In this study, we compared a conventional solid vitamin premix (SVP) and a liquid nanoscale vitamin premix (LNVP) under simulated storage, thermal treatment, gastrointestinal digestion, dialysis, and fecal fermentation conditions. The changes in vitamin contents were evaluated using chromatographic analysis, and vitamin retention, apparent disappearance, and dialysable fractions were calculated based on vitamin mass balance. Under the tested experimental conditions, LNVP showed different vitamin retention patterns compared with SVP during storage and thermal challenges, particularly for vitamins D and E. During simulated gastrointestinal processing, differences in vitamin recovery and dialysable fractions were observed between the two commercial formulations. These findings provide comparative information on the stability and in vitro behavior of different vitamin premix formats. Further in vivo studies using complete feed matrices are required to determine the nutritional significance of these differences in practical swine production.

## 1. Introduction

Vitamins, classified into fat-soluble (FSVs) and water-soluble (WSVs), are essential micronutrients for livestock [1]. Among the FSVs, vitamins A, D, and E act as critical regulators of diverse physiological functions ranging from muscle and skeletal growth to immune homeostasis and reproduction, and their dietary supply in intensive swine production relies heavily on formulated feeds [2,3,4]. As vitamin concentrations in complete feed are almost exclusively determined by premix inclusion rates, precise control and routine monitoring of vitamin content are imperative to maximize animal health and production performance, while avoiding unnecessary supplementation and the associated costs or toxicological risks [5,6,7].

However, FSVs in conventional solid vitamin premixes (SVPs) are highly sensitive to environmental stressors due to their chemical structures, which are rich in unsaturated double bonds [8,9]. This instability is further compounded by the physical heterogeneity and uneven distribution inherent to SVPs [1,10]. Notably, vitamin A in conventional solid premixes has been reported to decrease substantially during storage at elevated temperatures, whereas vitamin E oxidation can be accelerated by moisture and redox-active trace minerals [8]. Furthermore, FSVs are also exposed to acidic gastric conditions and digestive processes that may alter their chemical stability and transfer to the proximal intestine [11,12]. These challenges can be further compounded during feed processing and may potentially contribute to the adoption of over-fortification strategies [13].

Compared with conventional solid matrices, nano-encapsulation technologies may improve the physical protection and dispersibility of bioactive compounds [14]. Liquid nanoscale vitamin premixes (LNVPs) typically adopt an oil-in-water delivery structure, in which hydrophobic FSVs are embedded in a lipid phase and stabilized by an interfacial layer [15]. The small particle size increases the surface-area-to-volume ratio and may improve vitamin dispersibility and kinetic stability in aqueous systems [16,17]. The interfacial layer may also reduce the exposure of encapsulated vitamins to oxygen and other environmental stressors, while nanoscale delivery systems may alter interactions with digestive components that influence FSV transfer [17,18]. Despite these potential advantages, comparative evaluations of LNVPs and conventional SVPs in terms of storage stability, pelleting thermal tolerance, and simulated porcine gastrointestinal conditions remain limited.

Accordingly, this study was designed to systematically evaluate the relative stability and in vitro gastrointestinal transfer of vitamins A, D, and E in LNVPs compared with conventional SVPs. Because the two commercial premixes differed in composition, declared vitamin concentrations, physical form, and dosing basis, all core comparisons were normalized to the experimentally measured initial vitamin content within each formulation and are interpreted as relative formulation-specific differences. Physicochemical characterization included microscopy-based morphological observation, particle size analysis, and qualitative assessment of aqueous dispersion uniformity. Stability was assessed during 40-day storage at different temperatures and under simulated high-temperature pelleting conditions. A simulated porcine gastrointestinal digestion model was used to determine an intestinal dialysable fraction as an operational in vitro transfer endpoint, and a subsequent fecal fermentation assay was used to assess vitamin behavior under simulated colonic microbial conditions. We hypothesized that LNVPs would exhibit superior relative vitamin retention during storage and thermal processing, as well as higher dialysable fractions after simulated intestinal digestion, than SVPs under the tested conditions.

## 2. Materials and Methods

### 2.1. Sources and Composition of Premixes

The solid vitamin premixes (SVPs) and liquid nanoscale vitamin premixes (LNVPs) were provided by a collaborative enterprise. SVPs are commercial multi-vitamin premixes specifically formulated for swine feed. LNVPs were fabricated via oil-in-water nanoemulsion encapsulation technology (patent No. CN202410925012.9). Specific manufacturing parameters, excipient composition, and other formulation details were not available to the authors due to commercial confidentiality. The declared composition of the premixes is shown in Table 1.

### 2.2. Microstructural and Particle Size Analysis

To characterize the structural features of the two premixes, SVP and LNVP samples were placed on glass slides and observed under a digital microscope (Olympus BX51; Olympus Optical Co., Ltd., Tokyo, Japan). For scanning electron microscopy, samples were fixed in 2.5% glutaraldehyde, dehydrated through a graded ethanol series, and then dried with CO_2_ using a critical point dryer (Quorum K850; Quorum Technologies Ltd., Lewes, UK). The dried samples were mounted on specimen stubs, sputter-coated with a gold-palladium layer using a sputter coater (HITACHI MC1000, Hitachi, Japan), and viewed under a scanning electron microscope (Hitachi Regulus 8100, Hitachi, Tokyo, Japan) at an accelerating voltage of 10 kV [19]. For transmission electron microscopy, a drop of sample suspension was placed on a carbon-coated copper grid, allowed to settle at room temperature for 5 min, blotted with filter paper, and negatively stained with 2% phosphotungstic acid for 60 s. After air-drying at room temperature, the grid was imaged using a transmission electron microscope (HT7800, Hitachi, Tokyo, Japan) at an accelerating voltage of 80 kV [20]. Because negative staining and drying may affect the morphology of colloidal systems, transmission electron microscope-derived particle dimensions were interpreted as image-based morphological measurements under the applied preparation conditions rather than as direct measurements of native hydrated droplet size [20,21]. Image-based particle measurements were performed using ImageJ software (version 1.54g, National Institutes of Health, Bethesda, MD, USA). The maximum Feret diameter of SVPs was quantified from scanning electron microscopy images, and the particle size range of LNVPs was derived from transmission electron microscopy images. For both formulations, 300 particles were measured across three independent fields of view.

### 2.3. Dispersibility Test

To evaluate compatibility with aqueous delivery systems, the dispersibility of SVPs and LNVPs was assessed across five concentration ratios. For SVPs, premix-to-ultrapure water mass-to-volume ratios (*w*/*v*) of 1:9, 2:8, 3:7, 4:6, and 5:5 were prepared; for LNVPs, corresponding premix-to-water volume ratios (*v*/*v*) were used. The tubes were vortex-mixed at 1000 rpm for 1 min and then centrifuged at 654× *g* for 10 min [22]. A qualitative assessment based on visual appearance of each sample was recorded immediately after centrifugation to assess dispersion and sedimentation behavior.

### 2.4. Long-Term Storage and Thermal Stability Assays

For storage stability, SVPs and LNVPs were dispensed into 15 mL centrifuge tubes in triplicate (three independent sample tubes per storage condition). The tubes were sealed with parafilm, then wrapped sequentially in aluminum foil and two layers of black plastic bags to ensure light exclusion, and stored in temperature-controlled incubators at 4 °C, 25 °C, and 40 °C for 40 days. Samples were collected on days 0, 5, 10, 15, and 40 to determine the retention rates of vitamins A, D, and E [23].

To simulate the thermal stress associated with feed pelleting without the confounding effects of actual pellet formation, samples (10 g of SVPs or 10 mL of LNVPs) were incubated at 65 °C for 30 min or 85 °C for 3 min [9,24]. These temperature–time regimes were selected to match the range of conditioning temperatures and retention times commonly reported for commercial swine feed pelleting operations, and are widely used as standardized acute thermal stress models in feed vitamin stability research. This design enables isolated assessment of the pure thermal effect on vitamin degradation, independent of confounding factors including moisture, mechanical pressure and shear friction. Samples were then cooled to room temperature (RT) for vitamin content determination.

### 2.5. In Vitro Evaluation of Porcine Gastrointestinal Digestion, Dialysability, and Fecal Fermentation

#### 2.5.1. Simulated Porcine Gastric and Intestinal Digestion

A sequential in vitro digestion and fermentation procedure was conducted to evaluate the behavior of vitamins A, D, and E under simulated porcine gastrointestinal and subsequent large-intestinal conditions [25,26,27]. The experiment was conducted using independent parallel assays for each digestion stage to avoid variation caused by repeated sampling and volume loss with modifications. Briefly, 0.5 g of SVP or 0.5 mL of LNVP was added to a 250 mL Erlenmeyer flask and mixed thoroughly with 25 mL of 0.1 M phosphate buffer and 10 mL of 0.2 M HCl (pH 2.0). Then, 1 mL of porcine pepsin solution (20 g/L, Aladdin, Shanghai, China) was added to each flask. The mixture was incubated at 39 °C with continuous shaking (50 rpm) for 2 h to simulate gastric digestion. Following gastric hydrolysis, 10 mL of 0.2 M phosphate buffer and 5 mL of 0.6 M NaOH were added sequentially to adjust the mixture to pH 6.8. Subsequently, 1 mL of porcine pancreatin solution (100 g/L, Aladdin, Shanghai, China) was added, and the mixture was incubated at 39 °C with shaking for 4 h to simulate small-intestinal digestion.

#### 2.5.2. Dialysis of Intestinal Digestate and Collection of the Post-Dialysis Retentate

After intestinal digestion, the entire volume of digesta (approximately 52 mL) was transferred into a 10 kDa molecular weight cutoff (MWCO) dialysis tubing (22 mm flat width, Cat. No. 68100, Thermo Scientific™, Waltham, MA, USA). The dialysis membrane containing the digested sample was immersed in 100 mL phosphate-buffered saline (PBS) and incubated at 37 °C for 6 h with orbital shaking at 50 rpm. The external PBS was not replaced during the dialysis period. After dialysis, the external PBS (dialysate) and the residual liquid inside the tubing (retentate) were collected separately. The vitamin content of the dialysate was quantified to determine the dialysable fraction under the specified in vitro conditions. The post-dialysis retentate was subsequently used for fecal fermentation.

#### 2.5.3. Fecal Fermentation of the Post-Dialysis Retentate

A subsequent in vitro fecal fermentation was performed using the post-dialysis retentate generated from the preceding gastrointestinal digestion and dialysis steps. Fresh fecal samples were collected from three healthy growing Landrace × Yorkshire pigs and immediately placed in pre-warmed (39 °C) anaerobic thermos flasks and transported to the laboratory. The mineral and buffering components of the fermentation medium were prepared according to the formulation, with modifications in the inoculum source and fermentation setup for use with porcine fecal material [26]. For inoculum preparation, the pooled feces were homogenized with pre-warmed buffer solution at a dilution ratio of 1:20 (*w*/*v*). The suspension was filtered through four layers of sterile gauze to remove coarse particles, and the filtrate was maintained anaerobically at 39 °C under continuous CO_2_ saturation. A 10 mL aliquot of the post-dialysis retentate was transferred into 160 mL fermentation bottles containing 5 mL fecal inoculum and 35 mL anaerobic fermentation medium, resulting in a final fermentation volume of 50 mL. The vessels were incubated anaerobically at 39 °C with continuous shaking for 48 h. Three independent experimental trials were conducted, with five parallel vessels per formulation in each trial. Samples were collected at 0, 12, 24, and 48 h after fermentation initiation. The collected samples were immediately centrifuged, and the supernatants were stored at −20 °C until vitamin determination. The disappearance rates were calculated based on the total vitamin amount remaining in the entire dialysed digestion system rather than the aliquot used for fermentation.

### 2.6. Analysis of Vitamins A, D, and E

The concentrations of vitamins A, D, and E in the samples were determined using high-performance liquid chromatography with UV detection (HPLC-UV; Thermo Scientific Ultimate 3000, Waltham, MA, USA) based on the Chinese national standard methods for vitamin determination in feed [28,29,30]. Because the samples collected in the present study were liquid samples from different experimental stages rather than conventional feed samples, the sample volume and extraction solvent volume were adjusted accordingly. Briefly, 1 mL of each sample was transferred into a centrifuge tube and mixed with 9 mL of methanol. The mixture was vortex-mixed thoroughly and subjected to ultrasonic extraction at 45 °C for 30 min, followed by cooling to room temperature. The extracts were thoroughly mixed and filtered through a 0.22 μm organic filter—phase membrane filter before HPLC analysis. Chromatographic separation was performed on a C_18_ column (150 mm × 4.6 mm, 5 μm particle size; Thermo Fisher Scientific, Waltham, MA, USA) at a column temperature of 25 °C. The mobile phase consisted of methanol–water (98:2, *v*/*v*) with isocratic elution at a flow rate of 1.0 mL/min. The injection volume was 10 μL. Detection wavelengths were set at 325, 265 and 294 nm for vitamins A, vitamin D_3_ and vitamin E, respectively. Quantification was performed using an external-standard method, with all reference standards purchased from Solarbio (Beijing, China): retinyl acetate (vitamin A, Cat. No. SV8061), cholecalciferol (vitamin D_3_, Cat. No. IC0360) and dl-α-tocopheryl acetate (vitamin E, Cat. No. IYT100694). Calibration curves for each compound were constructed using a series of working solutions at different concentrations, and the vitamin content in each sample was calculated from the corresponding chromatographic peak area.

### 2.7. Calculation of Vitamin Retention, Disappearance, Dialysable Fraction, and Cumulative Apparent Disappearance

Based on the quantified concentrations, the retention and disappearance rates of the vitamins were calculated as previously described [31,32]. The initial vitamin content within each formulation was defined as the baseline (100% retention and 0% disappearance), and subsequent measurements were expressed as the relative proportion of residual vitamins and disappearance over the incubation period, respectively [24].

For storage stability and thermal tolerance experiments, vitamin retention was calculated as follows:

$$Retention\;rate\;(\%)=(M_{t}\;/M_{0})\times 100$$

$$Apparent\;disappearance\;rate\;\left(\%\right)=[(M_{0}-M_{t})/M_{0}]\times 100$$

where *M*_0_ represents initial measured vitamin content and *M_t_* represents measured vitamin content at the end of the specific stage. The term “apparent disappearance” refers to the reduction in measured vitamin amount relative to the initial value. This parameter reflects the overall change in measurable vitamin content and does not distinguish among chemical degradation, structural transformation, release from carrier matrices, or analytical recovery differences.

During simulated gastrointestinal digestion, the initial vitamin amount introduced into each digestion system was defined as $M_{0}$. After each digestion stage, the total vitamin amount recovered from the corresponding digestion fraction was calculated.

$$M_{t}=C_{t}\times V_{t}$$

where *C_t_* represents measured vitamin concentration after the specific digestion stage, *V_t_* represents total volume of the corresponding digestion system, *M_t_* represents recovered vitamin amount after digestion.

The apparent disappearance after each digestion stage was calculated as:

$$Stage\;apparent\;disappearance\;\left(\%\right)=[(M_{0}-M_{t})/M_{0}]\times 100$$

This value represents the reduction in measurable vitamin amount during each simulated gastrointestinal stage. It may include vitamin release from the formulation matrix, redistribution among different phases, enzymatic transformation, and loss of detectable vitamin compounds.

The intestinal dialysable fraction was calculated separately as an operational endpoint of membrane transfer during dialysis.

$$M_{t}=C_{d}\times V_{d}$$

$$Dialysable\;fraction\;\left(\%\right)=M_{d}/M_{0}\times 100$$

Here, *C_d_* is vitamin concentration measured in the external dialysis solution after dialysis, *V*_d_ is volume of external dialysis medium, *M_d_* is total vitamin amount recovered in the dialysate, and *M*_0_ is initial vitamin amount introduced into the simulated gastrointestinal digestion system. The dialysable fraction represents the proportion of the initial vitamin amount recovered in the external dialysis medium under the specified in vitro dialysis conditions. It is an operational measurement of membrane transfer and should not be interpreted as direct intestinal absorption or in vivo bioaccessibility.

For the fecal fermentation stage, vitamin disappearance was calculated relative to the vitamin amount present in the post-dialysis retained fraction before fermentation:

$$Cumulative\;apparent\;disappearance\;\left(\%\right)=\frac{\left[M_{0}\;-\left(C_{fer}\times 50\right)\times \;\frac{V_{r}}{10}\right]}{M_{0}}\times 100$$

$$Intestinal\;non-dialysable\;fraction\left(\%\right)=disappearance\;rate\;\left(\%\right)-dialysable\;fraction\;(\%)$$

Here, *C*_fer_ is vitamin concentration after fermentation, *V*_r_ is total post-dialysis retentate volume, 10 denotes the volume of the retained liquid used for fermentation, and 50 denotes the final fermentation volume (mL). This value represents the apparent change in vitamin amount during the fecal fermentation stage and was not interpreted as microbial degradation alone.

### 2.8. Statistical Analysis

All data were analyzed using SPSS 26.0 statistical software (IBM Corp., Chicago, IL, USA). For storage stability, two-way ANOVA was used to evaluate the main effects of formulation type (SVP vs. LNVP), storage time, and their interaction within each storage-temperature condition. Pairwise comparisons between SVP and LNVP at individual storage time points were performed using Student’s *t*-test. For the high-temperature stress experiment, simulated gastrointestinal digestion assay, intestinal dialysability analysis, and fecal fermentation assay, comparisons between SVP and LNVP were conducted using Student’s *t*-test. A value of *p* < 0.05 was considered statistically significant, and 0.05 ≤ *p* < 0.10 was considered a statistical trend. Data were plotted using GraphPad Prism version 8.0.1 (GraphPad Software, San Diego, CA, USA) and expressed as mean ± standard deviation (SD). Adobe Illustrator 2020 (Adobe, San Jose, CA, USA) was used for image layout and formatting.

## 3. Results

### 3.1. Morphological and Dispersibility Characteristics of SVPs and LNVPs

**Figure 1:**
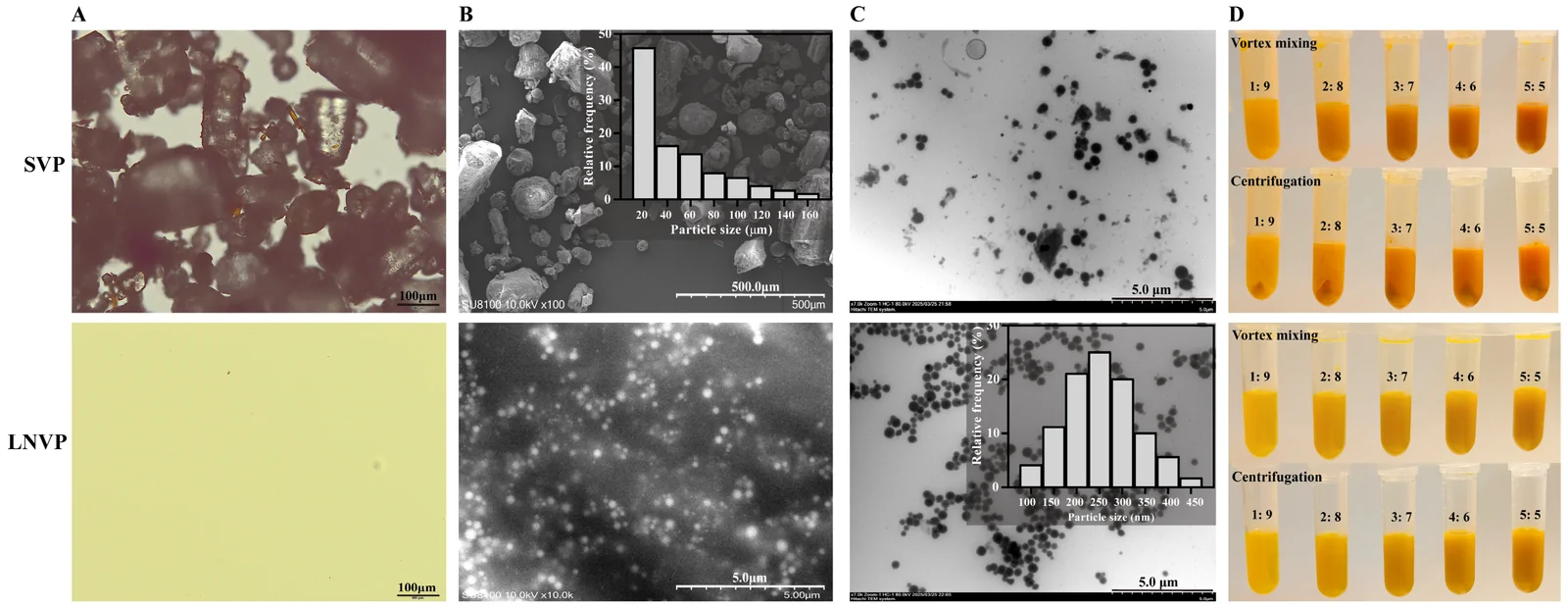
Morphological characterization of solid vitamin premix (SVP) and liquid nanoscale vitamin premix (LNVP). (**A**) Optical microscopy; (**B**) scanning electron microscopy; (**C**) transmission electron microscopy; (**D**) qualitative dispersion uniformity test. Scanning electron microscopy was used to determine Feret diameters of irregular micron-sized SVP particles, whereas transmission electron microscopy was applied for nanoscale LNVP droplets due to high resolution and low risk of particle deformation.

The microstructural and dispersion uniformity characteristics of SVPs and LNVPs are shown in Figure 1. As illustrated in Figure 1A–C, SVPs appeared as irregular solid particles with rough surfaces and a heterogeneous size distribution (20–160 μm), whereas LNVPs exhibited a homogeneous and transparent liquid matrix containing uniformly distributed spherical nanoparticles (100–450 nm). In terms of aqueous dispersibility (Figure 1D), SVPs exhibited macroscopic partial precipitation upon initial contact with water and clear phase separation following centrifugation. Conversely, LNVPs had a visually consistent appearance and remained predominantly free of sediment across the evaluated concentration gradients, even when exposed to mechanical agitation and significant centrifugal forces, indicating good dispersion stability under the tested conditions.

### 3.2. Retention Rates of FSVs Under Different Storage Temperatures

**Figure 2:**
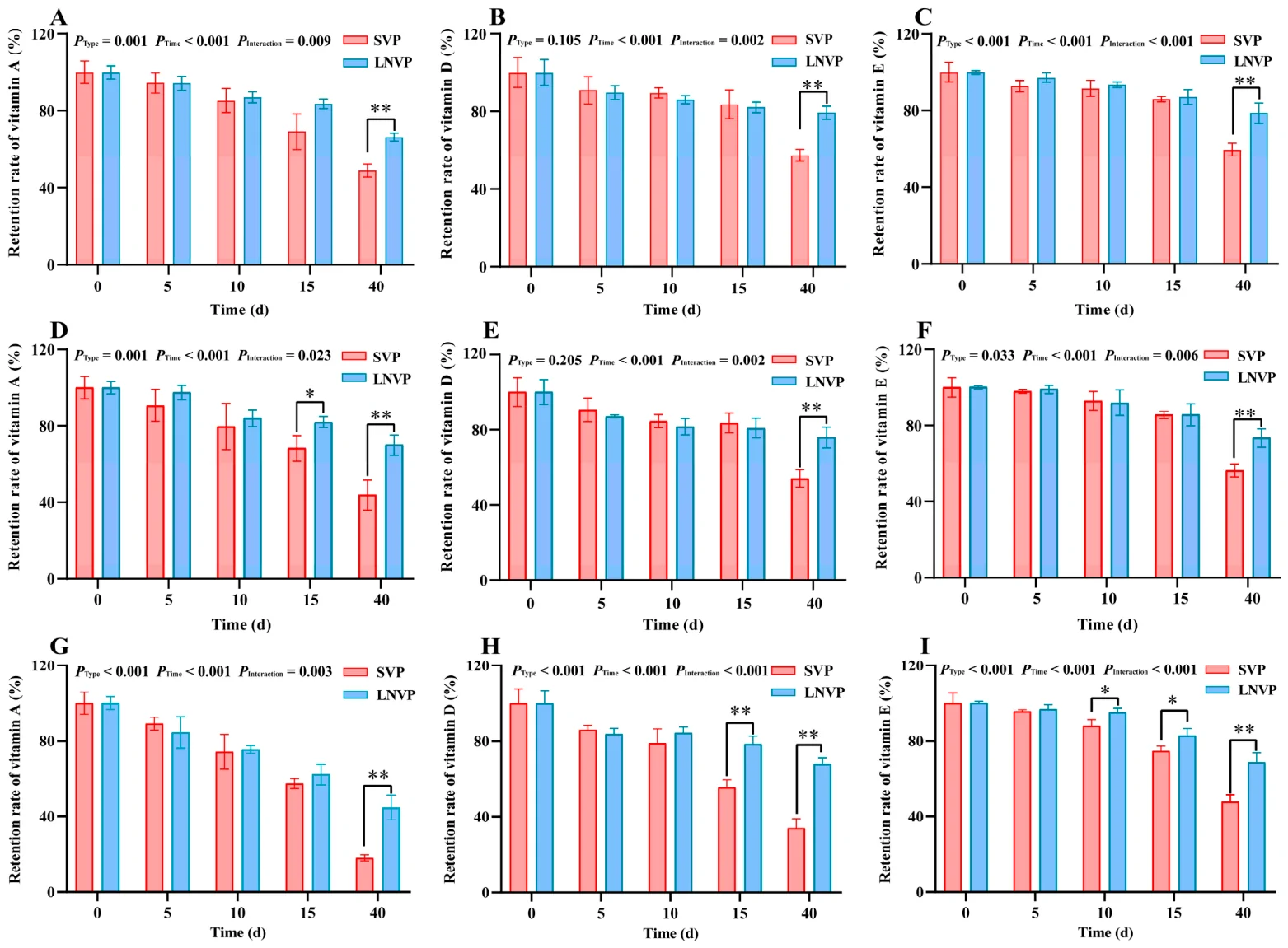
Effect of storage conditions on retention rates of solid vitamin premix (SVP) and liquid nanoscale vitamin premix (LNVP). (**A**–**C**) Vitamin retention during storage at 4 °C; (**D**–**F**) vitamin retention during storage at 25 °C; and (**G**–**I**) vitamin retention during storage at 40 °C. * and ** indicate significant differences between SVP and LNVP at the same time point (*p* < 0.05 and *p* < 0.01).

The retention rates of vitamins A, D, and E in both SVPs and LNVPs declined progressively with prolonged storage and increasing temperature (Figure 2). Formulation type, storage time, and their interaction were significant factors affecting relative vitamin retention. Both groups maintained high relative vitamin retention during storage at 4 °C, with LNVPs showing significantly greater relative retention rates than SVPs by day 40 (*p* < 0.01, Figure 2A–C). At 25 °C, the discrepancy in relative retention between LNVPs and SVPs widened to 10–15% by day 40 (Figure 2D–F). Notably, under accelerated thermal stress at 40 °C, LNVPs exhibited greater thermal resilience and consistently showed significantly higher relative retention of all three FSVs than SVPs at the 40-day time point (*p* < 0.01, Figure 2G–I). In both formulations, vitamin retention rates declined progressively as the storage temperature increased. Collectively, the storage stability results demonstrate that the liquid nanoscale matrix provides more effective protection against temperature-induced degradation of fat-soluble vitamins.

### 3.3. Retention Rates of FSVs Under High-Temperature Conditions

**Figure 3:**
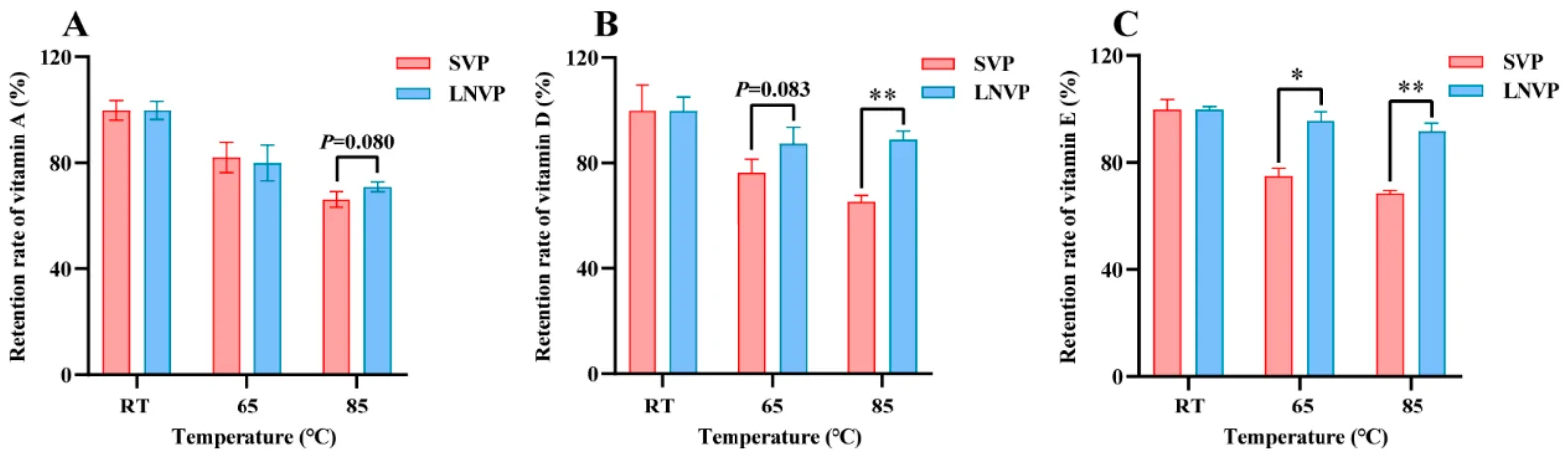
Fat-soluble vitamin retention rates of solid vitamin premix (SVP) and liquid nanoscale vitamin premix (LNVP) under high-temperature tests. Premix samples were exposed to room temperature (RT, untreated control), 65 °C for 30 min, or 85 °C for 3 min to simulate varying degrees of acute high-temperature stress encountered during commercial feed processing. (**A**) Vitamin A, (**B**) vitamin D, and (**C**) vitamin E. * and ** indicate significant differences between SVP and LNVP at the same temperature (*p* < 0.05 and *p* < 0.01).

The retention rates of vitamins A, D, and E in both SVPs and LNVPs exhibited significant temperature sensitivity, characterized by a progressive decline as the treatment temperature rose from 65 °C to 85 °C (Figure 3). At 65 °C, LNVPs showed a trend toward higher vitamin D retention (*p* = 0.083) and maintained significantly higher vitamin E retention (*p* < 0.05) than SVPs. At 85 °C, the retention rates of vitamins D and E were significantly higher in LNVPs (*p* < 0.01). No significant difference in vitamin A retention rate was observed between SVPs and LNVPs at either 65 °C or 85 °C, although a statistical trend toward higher vitamin A retention in LNVPs was observed at 85 °C (*p* = 0.080).

### 3.4. Stability of FSVs During In Vitro Simulated Porcine Gastrointestinal Digestion and Fecal Fermentation

**Figure 4:**
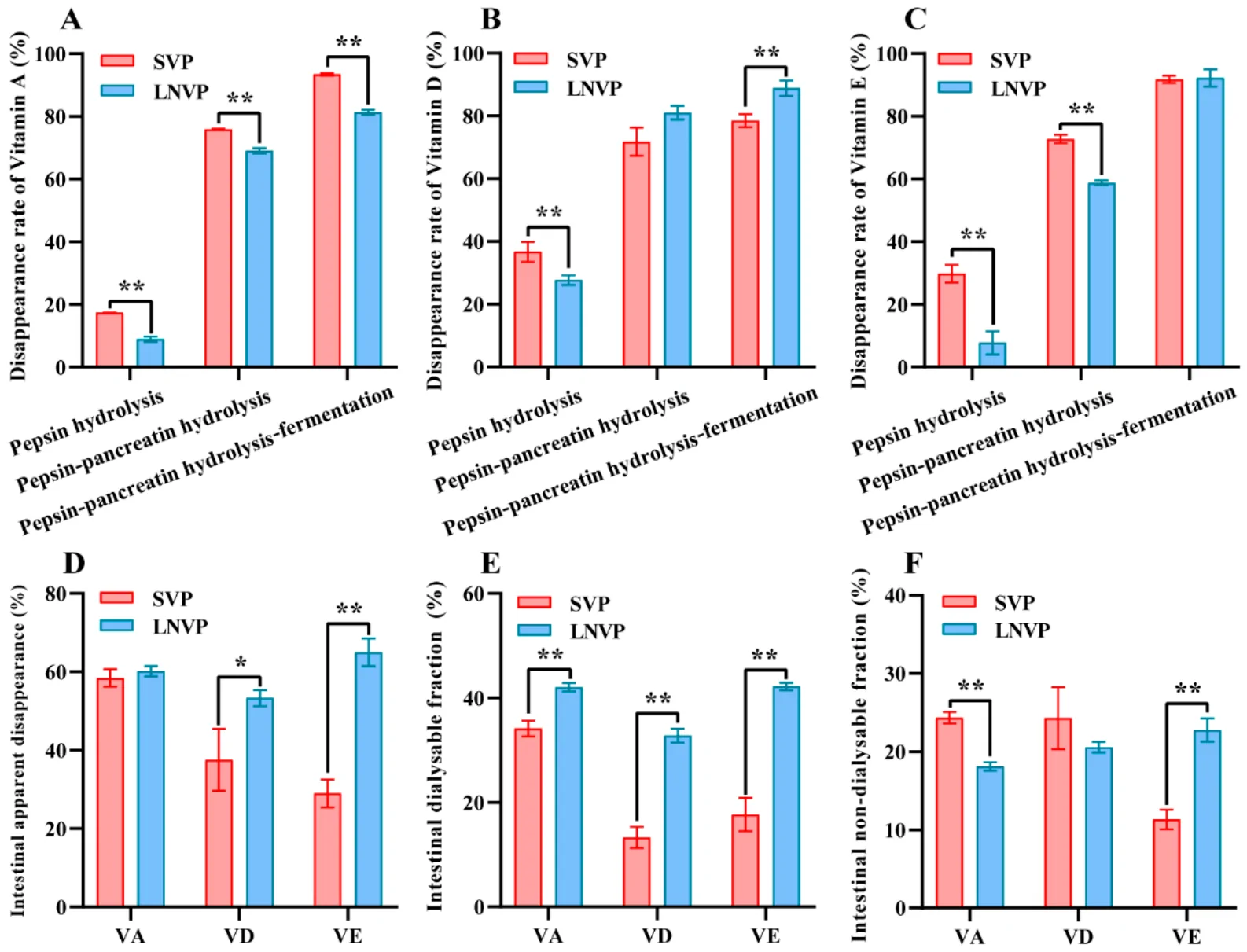
Apparent disappearance and dialysability of vitamins A, D, and E during simulated gastrointestinal digestion and subsequent fecal fermentation. (**A**–**C**) Cumulative apparent disappearance rates of vitamin A (VA), vitamin D (VD), and vitamin E (VE) after gastric digestion, intestinal digestion, and subsequent fecal fermentation, respectively. (**D**–**F**) Intestinal apparent disappearance rate, dialysable fraction, and non-dialysable fraction, respectively. * and ** indicate significant differences between SVP and LNVP at the same time point (*p* < 0.05 and *p* < 0.01).

To investigate the gastrointestinal behavior of vitamins in different premix formulations, SVPs and LNVPs were subjected to sequential simulated gastrointestinal digestion, dialysis, and fecal fermentation. The disappearance rates were calculated based on the reduction in vitamin concentrations detected in the corresponding fractions relative to the initial vitamin input. The apparent disappearance rates of vitamins A, D, and E progressively increased after sequential simulated gastrointestinal digestion and fecal fermentation (Figure 4A–C). Across the measured conditions, vitamin A showed a consistently higher disappearance rate in SVPs than in LNVPs (*p* < 0.01). For vitamin D, SVPs showed a substantially higher disappearance rate after gastric pepsin digestion, but a markedly lower rate in the fecal fermentation stage (*p* < 0.01). Similarly, SVPs had a notably greater cumulative disappearance rate of vitamin E during the gastric digestion stage relative to LNVPs (*p* < 0.01). In the simulated intestinal phase, the disappearance rates of vitamins D and E were significantly higher in LNVPs (Figure 4D). Correspondingly, the intestinal dialysable fractions of all three FSVs were higher in LNVPs (*p* < 0.01, Figure 4E). Furthermore, the intestinal non-dialysable fraction of vitamin A was lower in LNVPs than in SVPs, whereas that of vitamin E was higher in LNVPs (*p* < 0.01, Figure 4F).

The cumulative disappearance rate at each endpoint was calculated relative to the initial vitamin content of the corresponding experimental system. Gastric digestion, intestinal digestion, and fecal fermentation endpoints were generated from independent experimental sets to avoid changes in reaction volume caused by repeated sampling. Dialysis was incorporated into the intestinal-stage procedure. Following dialysis, a 10-mL aliquot of the post-dialysis retentate was used for fecal fermentation. The dialysable fraction represents the proportion of vitamin transferred to the external dialysis phase under the specified experimental conditions and should not be interpreted as direct in vivo intestinal absorption.

## 4. Discussion

The present study compared the relative stability and in vitro gastrointestinal transfer characteristics of SVPs and LNVPs, with particular emphasis on storage stability, acute thermal stress, and simulated porcine digestion. LNVPs were associated with greater visual dispersion stability, higher relative retention of vitamins A, D, and E during storage, higher retention of vitamins D and E under acute thermal stress, and higher dialysable fractions after simulated intestinal digestion. These findings support the potential application of liquid vitamin delivery systems for improving the physical handling and relative preservation of FSVs.

The morphological and dispersibility analyses revealed clear differences between the two premix formulations. SVPs exhibited irregular solid particles with a heterogeneous size distribution and visible agglomeration, whereas LNVPs showed a transparent liquid matrix containing uniformly dispersed spherical nanoparticles. Notably, the LNVPs system maintained excellent uniformity with no sediment formation after centrifugation. This superior dispersion uniformity is of critical importance for practical feed production, as homogeneous mixing of micronutrients is a core prerequisite to ensure consistent nutrient intake by animals [33]. The nanoscale morphology effectively inhibits particle aggregation, modulates the surface properties and particle size distribution of feed additives, and reduces interparticle van der Waals forces, thereby enhancing their dispersibility in aqueous systems [16,18,34]. However, the specific mechanisms responsible for these differences require further investigation.

The observed differences in storage stability and acute thermal tolerance suggest that the liquid delivery matrix may be associated with improved vitamin retention under the tested conditions [35]. Given that commercial vitamin premixes are susceptible to degradation during prolonged storage and high-temperature processing, evaluating their stability under storage and processing-related stress conditions is important [8,9]. In the present study, long-term storage and pelleting-simulated thermal exposure resulted in higher retention of vitamins D and E in LNVPs compared with SVPs under the evaluated conditions. Previous studies on encapsulated vitamin systems have reported that interfacial structures may improve the oxidative stability of sensitive compounds by reducing interactions with environmental pro-oxidants [36]. Meanwhile, this interfacial shell effectively isolates heat-labile vitamin molecules from direct high-temperature exposure, thereby retarding irreversible thermal degradation reactions [9,37]. Therefore, the higher vitamin retention observed in LNVPs may be associated with differences in formulation characteristics, including dispersion behavior and physical structure. However, because oxidative products, oxygen permeability, and detailed degradation pathways were not determined in the present study, the specific mechanisms underlying the improved retention of LNVPs remain to be elucidated. Collectively, these findings support an association between formulation type and vitamin retention performance, and are consistent with nano-encapsulation providing a potential protective effect against long-term storage deterioration and thermal processing damage.

The gastrointestinal fate of FSVs is influenced by their physicochemical properties, formulation characteristics, and interactions with digestive components [38,39]. In the present study, LNVPs and SVPs showed different vitamin disappearance patterns and dialysable fractions during simulated porcine gastrointestinal digestion. These differences indicate that formulation structure may influence vitamin behavior under in vitro gastrointestinal conditions. However, the observed differences should be interpreted as formulation-associated responses rather than direct evidence of enhanced intestinal absorption. During simulated gastric digestion, LNVPs showed lower apparent disappearance rates of vitamins A, D, and E. The nanoscale morphology and improved dispersion characteristics observed for LNVPs may be associated with a more homogeneous distribution of vitamin components within the digestion system [40,41]. Following intestinal digestion, LNVPs showed higher disappearance rates for vitamins D and E and higher dialysable fractions for all three FSVs. Apparent disappearance represents the net change in vitamin concentration within the experimental system and may include multiple processes, such as release from the formulation, degradation, redistribution, or transfer between fractions [1,42,43]. Similarly, the dialysable fraction reflects the proportion of vitamin transferred through the dialysis membrane under the defined experimental conditions rather than physiological uptake by intestinal epithelial cells [12,15,44]. Therefore, higher disappearance does not necessarily indicate greater degradation or improved utilization, and the higher dialysable fractions observed for LNVPs suggest altered vitamin transfer behavior during simulated intestinal digestion but do not demonstrate enhanced vitamin absorption in pigs.

Overall, the present findings demonstrate formulation-associated differences in vitamin retention, disappearance, and dialysability during simulated gastrointestinal processing. These results provide comparative information regarding the behavior of two commercial vitamin premix formulations under controlled in vitro conditions. However, further studies incorporating more physiologically representative digestion systems and animal validation are required to determine their relevance to vitamin utilization in swine nutrition.

Despite these promising findings, several limitations must be acknowledged. First, this study compares two commercial formulations of different compositions, and the observed performance differences cannot be unequivocally attributed solely to the nanoscale structure. Differences in excipient composition, antioxidant systems, or other proprietary formulation components may also contribute. Second, the reported particle size range was estimated from microscopy images and full physicochemical characterization of the nanoparticle systems remains incomplete. Full physicochemical characterization of the nanoparticle systems (DLS, PDI, zeta potential) was not performed, and particle size evolution during storage and digestion was not monitored. Third, vitamin measurements were obtained from sampled fractions rather than complete recovery of all phases of the experimental systems. Consequently, the study does not establish a complete whole-system vitamin mass balance, and the observed apparent disappearance cannot unambiguously distinguish chemical degradation from physical processes. Fourth, the dialysis endpoint only reflects dialysability under the specific assay conditions, and cannot be interpreted as a direct measure of intestinal bioaccessibility. Furthermore, all in vitro evaluations were conducted using pure vitamin premixes without a complete feed matrix to isolate and compare their intrinsic structural resilience. Therefore, further studies incorporating more physiologically representative digestion systems and animal validation are required to determine their relevance to vitamin utilization in swine nutrition.

## 5. Conclusions

The present study compared the physicochemical characteristics, storage stability, thermal tolerance, simulated gastrointestinal behavior, and fecal fermentation responses between SVP and LNVP under specific experimental conditions. The LNVP showed distinct physicochemical characteristics, including nanoscale morphology and improved dispersion behavior, and exhibited higher retention of vitamins A, D, and E under the evaluated storage and thermal treatment conditions. During simulated gastrointestinal digestion, the LNVP showed formulation-associated differences in vitamin disappearance and higher dialysable fractions of vitamins A, D, and E under the applied experimental conditions. These findings provide comparative evidence regarding the behavior of the two tested commercial vitamin premixes during storage, thermal exposure, simulated digestion, and fecal fermentation. Further studies using complete-feed matrices and in vivo animal experiments are required to determine whether these formulation-associated differences translate into practical benefits in swine production.

## Figures and Tables

**Table 1:** The composition of vitamin premixes.

Vitamins ^1^	SVP Declared (Measured)	LNVP Declared (Measured)
Vitamin A, ×10^4^ IU/kg or IU/L	1000 (954.47)	500 (502.41)
Vitamin D, ×10^4^ IU/kg or IU/L	100 (92.41)	150 (156.15)
Vitamin E, IU/kg or IU/L	18,000 (16,746.88)	15,000 (14,195.39)
Vitamin B1, mg/kg or mg/L	2000	2400
Vitamin B2, mg/kg or mg/L	5000	1200
Vitamin B6, mg/kg or mg/L	1500	1800
Vitamin B7, mg/kg or mg/L	50	9

^1^ Vitamin sources: Vitamin A, retinyl acetate; Vitamin D_3_, cholecalciferol; Vitamin E, dl-α-tocopheryl acetate; Vitamin B_1_, thiamine hydrochloride; Vitamin B_2_, riboflavin; Vitamin B_6_, pyridoxine hydrochloride; Vitamin B_7_, biotin. SVP concentrations are expressed on a mass basis, whereas LNVP concentrations are expressed on a volume basis. Because the two formulations were not chemically matched, the results are interpreted as comparisons between the tested commercial products rather than as dose-equivalent comparisons. All core indicators in this study were calculated relative to the initial experimentally measured vitamin content within each formulation. Because the two commercial formulations were not compositionally identical and were administered on different mass- and volume-based scales, the data support relative formulation comparisons rather than direct comparisons of absolute vitamin delivery.

## Data Availability

The original contributions presented in this study are included in the article. Further inquiries can be directed to the corresponding author.
